# Adverse event rates and economic burden associated with purine nucleoside analogs in patients with hairy cell leukemia: a US population-retrospective claims analysis

**DOI:** 10.1186/s13023-020-1325-9

**Published:** 2020-02-13

**Authors:** Narendranath Epperla, Melissa Pavilack, Temitope Olufade, Richa Bashyal, Jieni Li, Shaum M. Kabadi, Huseyin Yuce, Leslie Andritsos

**Affiliations:** 1 Division of Hematology, The James Cancer Hospital and Solove Research Institute, The Ohio State University Comprehensive Cancer Center, A346 Starling Loving Hall, 320W 10th Ave, Columbus, OH 43210 USA; 2 AstraZeneca, Gaithersburg, MD USA; 3 STATinMED Research, Plano, TX USA; 4 New York City College of Technology, New York, NY USA; 5 Division of Hematology and Oncology, The University of New Mexico, Albuquerque, NM USA

**Keywords:** Hairy cell leukemia, Adverse events, Purine nucleoside analogs, Myelosuppression

## Abstract

**Background:**

Purine nucleoside analogs (PNAs) are the recommended first-line treatment for patients with hairy cell leukemia (HCL), but they are associated with adverse events (AEs). Due to a lack of real-world evidence regarding AEs that are associated with PNAs*,* we used commercial data to assess AE rates, AE-related health care resource utilization (HCRU), and costs among PNA-treated patients with HCL. Adults aged ≥18 years with ≥2 claims for HCL ≥30 days apart from 1 January 2006 through 31 December 2015 were included. Included patients had ≥1 claim for HCL therapy (cladribine ± rituximab or pentostatin ± rituximab [index date: first claim date]) and continuous enrollment for a ≥ 6-month baseline and ≥ 12-month follow-up period. Patient sub-cohorts were based on the occurrence of myelosuppression and opportunistic infections (OIs). Generalized linear models were used to compare HCRU and costs.

**Results:**

In total, 647 PNA-treated patients were identified (mean age: 57.1 years). Myelosuppression and OI incidence were 461 and 42 per 1000 patient-years, respectively. Adjusted results indicated that those with myelosuppression had higher rates of hospitalization (47.4% vs 12.4%; *P* < .0001) and incurred higher mean inpatient costs ($23,517 vs $12,729; *P* = .011) and total costs ($57,325 vs $34,733; *P* = .001) as compared with those without myelosuppression. Similarly, patients with OIs had higher rates of hospitalization (53.8% vs 30.8%; *P* = .025) and incurred higher mean inpatient costs ($21,494 vs $11,229; *P* < .0001) as compared with those without OIs.

**Conclusions:**

PNA therapy is highly effective but associated with significant toxicities that increase costs; these findings indicate a need for therapies with improved toxicity profiles and better risk stratification of patients at risk of developing myelosuppression and OIs.

## Background

Hairy cell leukemia (HCL) is a rare, chronic B-cell malignancy that comprises 8% of all lymphoproliferative disorders and 2% of all leukemias in the United States [1–4]. It is found to be 4–5 times more frequent among men than women, with ~ 1000 new cases reported in the United States each year [5]. The median age at HCL diagnosis is 55 years, and the cause is unknown [1, 6, 7].

HCL is currently incurable, but it is responsive to available therapies that restore hematopoiesis, resolve symptoms related to the underlying disease, and achieve sustained remission [3, 8]. Purine nucleoside analogs (PNAs; pentostatin and cladribine) are the recommended first-line treatments currently administered with or without the anti-CD20 antibody, rituximab [9–11]. PNAs have changed the natural history of this rare disease by achieving complete remission (CR) rates of approximately 70–90% with a median relapse-free survival of ~ 15 years [10, 11, 12]. Although PNA treatment improves CR rates, relapses are common, with rates of 34% with cladribine and 24% with pentostatin at 5 years, 42% with both agents at 10 years, and 48 and 47%, respectively, at 15 years [12]. According to the World Health Organization, the 5-year survival rate for HCL ranges from 78 to 92% [13].

Although they improve CR rates, PNA therapies are associated with clinically important adverse events (AEs), including prolonged myelosuppression and increased risk of infection [3]. Myelosuppression is one of the notable toxicities of both cladribine and pentostatin [14], and infection is one of the common causes of death among patients with HCL [15]. Moreover, both AEs have been associated with considerable health care burden [16]. Owing to the rarity of HCL, little is known about clinically significant AEs beyond clinical trial findings. Though previous studies have examined HCL’s health care burden, the burden of AEs among PNA-treated patients with HCL has not been investigated [17, 18]. Thus, using a large real-world claims database, we sought to (1) retrospectively examine the incidence and prevalence of AEs associated with PNA use, and (2) quantify the health care resource utilization (HCRU) and cost burden of PNA-treated HCL patients with incident myelosuppression and those with opportunistic infections (OIs).

## Methods

### Study design

This is a retrospective cohort study utilizing data from the IBM MarketScan® Commercial Claims and Encounters, Medicare Supplemental Database during the study period (1 January 2006 through 31 December 2016).

The MarketScan claims databases contain > 200 million unique patients since 1995, with data for > 77 million covered lives per data-year. The sample size is large enough to allow for the creation of a nationally representative data sample of Americans with employer-provided health insurance. The database comprises de-identified, person-specific health care data including clinical utilization, expenditures, insurance enrollment/plan benefit, inpatient, outpatient, and outpatient prescription information.

### Patient population

Patients included in the study were aged ≥18 years with ≥2 claims associated with an HCL diagnosis (International Classification of Diseases, Ninth Revision, Clinical Modification [ICD-9-CM] code: 202.4x, ICD-10-CM code: C91.4x) occurring ≥30 days apart during the study period (1 January 2006 through 31 December 2016). Patients had ≥1 prescription claim for HCL therapy (PNA as monotherapy or in combination with rituximab, ie, cladribine ± rituximab, pentostatin ± rituximab) during the identification period (1 July 2006 through 31 December 2015) following the first HCL diagnosis date. The first prescription claim date for an HCL therapy was defined as the index date. Patients were required to have continuous health plan enrollment with medical and pharmacy benefits for ≥6 months pre- and ≥ 12 months post-index date (baseline and follow-up period, respectively). Patients were excluded from the study if they had evidence of HCL therapy during the baseline period.

PNA-treated patients with HCL were further divided into sub-cohorts: (1) patients with and without myelosuppression; and (2) patients with and without OIs (conditions included in the OI definition are listed in Additional file 1: Table S1), to compare HCRU and costs between the groups.

The diagnostic conditions were identified using ICD-9-CM codes as listed in Additional file 1: Table S1 and Additional file 2: Table S2. All ICD-9-CM codes were mapped to ICD-10-CM codes based on the General Equivalence Mappings published by the Centers for Medicare & Medicaid Services [19].

### Study endpoints and definitions

Incidence and prevalence of AEs were assessed among all PNA-treated patients with HCL. HCRU and cost burden of PNA-treated patients were compared among HCL patients with and without incident myelosuppression and OIs.

AEs were selected based on a review of medical literature, clinical importance, and prescribing information for PNA therapies. Incidence was defined as the number of new cases of an AE during the follow-up period divided by the number of patients at risk (patients without any evidence of the AE in the 6-month baseline period). Prevalence was defined as the number of AEs (existing cases during the baseline period and new cases during the follow-up period) divided by the total number of patients. Incidence and prevalence were calculated as rates per 1000 patient-years. All-cause HCRU and costs were assessed among sub-cohorts of PNA-treated patients (those with and without myelosuppression, and those with and without OIs). All costs were adjusted to 2016 US $ (the last year of the study period) using the medical care component of the Consumer Price Index.

### Statistical analysis

Descriptive statistics on all study variables—including baseline, clinical characteristics, and outcome variables—were provided for PNA-treated patients with HCL. Statistical tests of significance (chi-squared for categorical and t-test for continuous variables) were conducted to assess differences between patients with and without myelosuppression and patients with and without OIs, separately. The level of significance was set at α = 0.05.

Generalized linear models (GLMs) were used to compare all-cause HCRU and cost outcomes between PNA sub-cohorts. The number of visits per patient was estimated using the negative binomial regression model. The proportion of patients with ≥1 visit was modeled using logistic regression, and cost data were examined using a GLM with log-link function and gamma variance. Cost data with a large proportion of zeros were examined using a two-part model in which the first part was a logistic regression of any use of service and the second part was a GLM regression of cost, conditional on the use of service. Covariates included in the GLM were age, sex, US region, Quan-Charlson comorbidity index (Quan-CCI) score, baseline symptoms and conditions, baseline individual comorbidities, baseline heme-related diagnoses, baseline medications, and baseline all-cause total health care costs. All the analyses were conducted using SAS® statistical software (Version 9.4, SAS Institute, Cary, NC, 2012). Neither Institutional Review Board approval nor consent was necessary for this study, as it was conducted with depersonalized claims data and does not contain any interactions with human participants or animals performed by any of the authors.

## Results

### Demographics and baseline characteristics

**Fig. 1:**
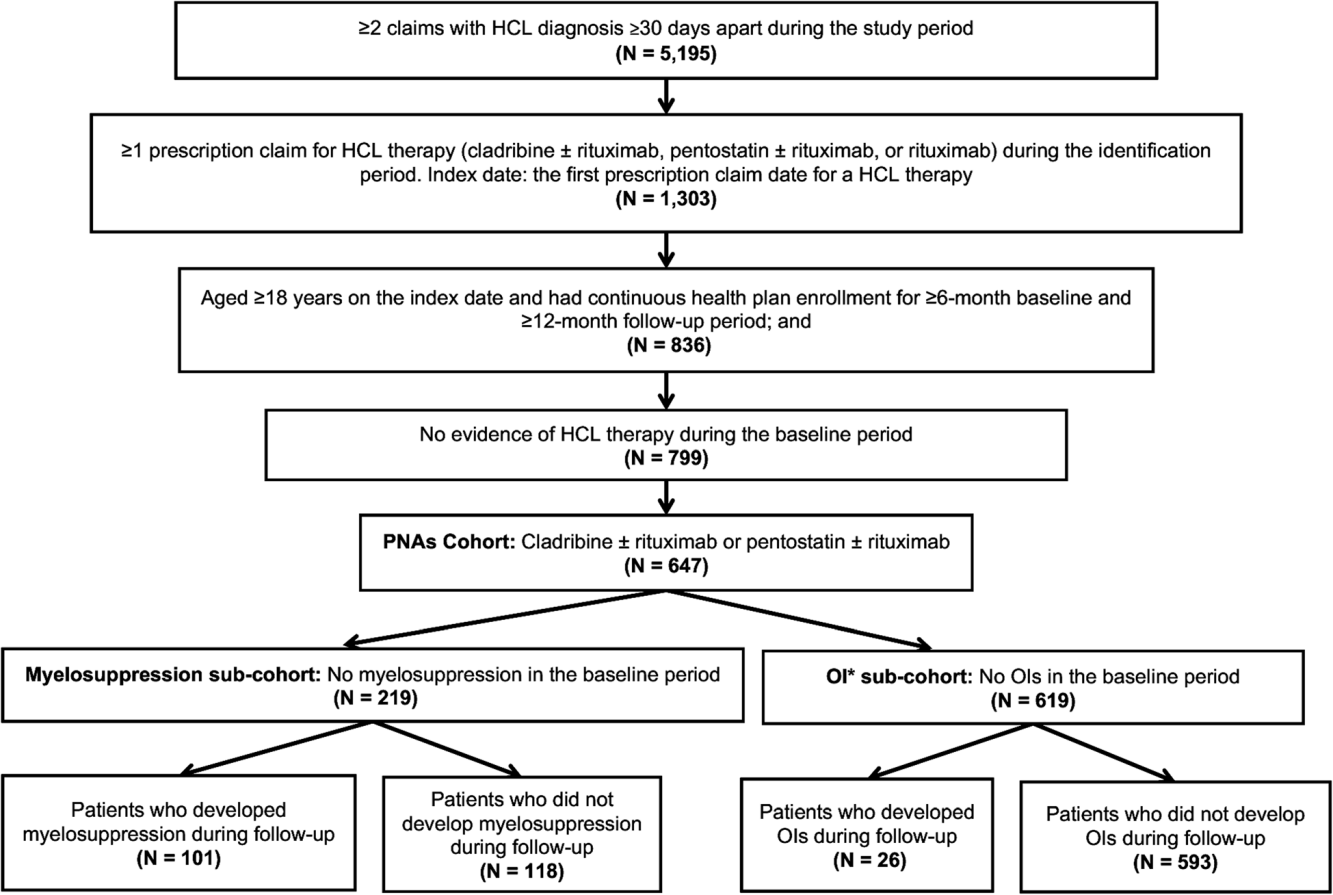
Patient selection criteria. ^*^OI was defined by any of the following conditions: pulmonary tuberculosis, atypical mycobacteria, cryptococcosis, aspergillosis, histoplasmosis, listeriosis, leishmaniasis, *Pneumocystis jiroveci* pneumonia, keratitis, onychomycosis, peritonitis, fungemia, endophthalmitis, septic/pyogenic arthritis, and osteomyelitis. *HCL* hairy cell leukemia, *OI* opportunistic infection

A total of 647 patients with HCL treated with PNAs (cladribine [*n* = 598]; pentostatin [*n* = 49]) met the inclusion criteria (Fig. 1). For the sub-cohort analysis, 219 patients did not have a diagnosis of myelosuppression in the baseline period, and 619 patients did not have a diagnosis of OI at baseline (Fig. 1).

The average age of PNA-treated patients with HCL was 57.1 years. Most of the patients were male (81.5%) and resided in the South US geographic region (32.1%). The average Quan-CCI score was 2.5. The most common comorbidities were aplastic anemia (38.2%), hypertension (30.4%), and diabetes (11.3%), and the most common coded symptoms in the baseline period were splenomegaly (29.4%), infectious complications (20.6%), and fatigue (17.3%) (Table 1).

**Table 1:** Demographic and baseline clinical characteristics of PNAs and sub-cohorts

Baseline characteristics	PNA-treated patients (*N* = 647)	Sub-cohort with no myelosuppression in baseline period (*N* = 219)			Sub-cohort with no OIs in baseline period (*N* = 619)		
		Without myelosuppression (*N* = 118)	With myelosuppression (*N* = 101)	*P*	Without OIs (*N* = 593)	With OIs (*N* = 26)	*P*
Mean age, years, mean ± SD	57.1 ± 12.2	61.0 ± 12.5	60.0 ± 14.9	.583	56.5 ± 12.0	61.9 ± 11.7	.026*
Sex, n (%)							
Male	527 (81.5)	97 (82.2)	77 (76.2)	.276	485 (81.8)	22 (84.6)	.714
Female	120 (18.5)	21 (17.8)	24 (23.8)	.276	108 (18.2)	4 (15.4)	.714
US geographic region, n (%)							
Northeast	117 (18.1)	17 (14.4)	23 (22.8)	.110	106 (17.9)	4 (15.4)	.745
North Central	172 (26.6)	33 (28.0)	27 (26.7)	.838	156 (26.3)	8 (30.8)	.614
South	208 (32.1)	38 (32.2)	24 (23.8)	.167	192 (32.4)	11 (42.3)	.291
West	129 (19.9)	27 (22.9)	24 (23.8)	.878	120 (20.2)	3 (11.5)	.277
Other	21 (3.2)	3 (2.5)	3 (3.0)	.847	19 (3.2)	0 (0.0)	.354
Comorbid conditions, mean ± SD							
Quan-CCI score	2.5 ± 1.3	2.6 ± 1.4	2.5 ± 1.1	.437	2.5 ± 1.3	3.1 ± 1.4	0.013*
Baseline symptoms/conditions, n (%)							
Infectious complications	133 (20.6)	28 (23.7)	22 (21.8)	.732	99 (16.7)	6 (23.1)	.396
Opportunistic infections	28 (4.3)	6 (5.1)	5 (5.0)	.964	0 (0.0)	0 (0.0)	NA
Pneumonia	55 (8.5)	15 (12.7)	7 (6.9)	.156	51 (8.6)	3 (11.5)	.603
Sepsis	11 (1.7)	0 (0.0)	3 (3.0)	.059	10 (1.7)	1 (3.8)	.415
Viral skin infection	4 (0.6)	1 (0.8)	0 (0.0)	.354	4 (0.7)	0 (0.0)	.674
Acute sinusitis	34 (5.3)	6 (5.1)	6 (5.9)	.782	33 (5.6)	1 (3.8)	.707
Chronic sinusitis	16 (2.5)	0 (0.0)	2 (2.0)	.125	15 (2.5)	1 (3.8)	.679
Abdominal pain	110 (17.0)	13 (11.0)	14 (13.9)	.523	104 (17.5)	3 (11.5)	.428
Fatigue	112 (17.3)	10 (8.5)	15 (14.9)	.139	101 (17.0)	7 (26.9)	.193
Splenomegaly	190 (29.4)	22 (18.6)	24 (23.8)	.354	174 (29.3)	11 (42.3)	.158
Baseline heme-related diagnosis, n (%)							
Aplastic anemia	247 (38.2)	41 (34.7)	47 (46.5)	.076	228 (38.4)	7 (26.9)	.236
ITP	9 (1.4)	0 (0.0)	0 (0.0)	NA	7 (1.2)	1 (3.8)	.239
AIHA	0 (0.0)	0 (0.0)	0 (0.0)	NA	0 (0.0)	0 (0.0)	NA
Other baseline individual comorbidities, n (%)							
Adenopathy	44 (6.8)	8 (6.8)	5 (5.0)	.568	41 (6.9)	1 (3.8)	.543
Diabetes	73 (11.3)	16 (13.6)	16 (15.8)	.634	61 (10.3)	7 (26.9)	.008*
Hypertension	197 (30.4)	33 (28.0)	33 (32.7)	.449	175 (29.5)	11 (42.3)	.164
Liver disease	33 (5.1)	1 (0.8)	2 (2.0)	.472	30 (5.1)	2 (7.7)	.553
Liver enlargement	13 (2.0)	0 (0.0)	2 (2.0)	.125	12 (2.0)	1 (3.8)	.526
Myocardial infarction	8 (1.2)	2 (1.7)	0 (0.0)	.189	7 (1.2)	1 (3.8)	.239
Renal failure	19 (2.9)	4 (3.4)	1 (1.0)	.236	15 (2.5)	1 (3.8)	.679

*AIHA* Autoimmune hemolytic anemia, *ITP* Idiopathic thrombocytopenia purpura, *OI* opportunistic infection, *PNA* purine nucleoside analog, *Quan-CCI* Quan-Charlson comorbidity index, *SD* Standard deviation

^*^Significant at *P* < .05

Findings on demographics and clinical characteristics of the sub-cohorts revealed no significant differences between patients with and without myelosuppression during the 6-month baseline period (Table 1). Only a few significant differences were observed among patients with and without OIs during the 6-month baseline period. Patients who developed OIs were older compared with those who did not (61.9 vs 56.5 years, *P* = .026). In addition, the mean Quan-CCI score was significantly higher among patients who developed OIs (3.1 vs 2.5, *P* = .013). Likewise, a significantly greater proportion of patients who developed OIs had diabetes in the baseline period (26.9% vs 10.3%; *P* = .008) (Table 1).

### Incidence and prevalence of AEs among overall PNA-treated patients with HCL

**Fig. 2:**
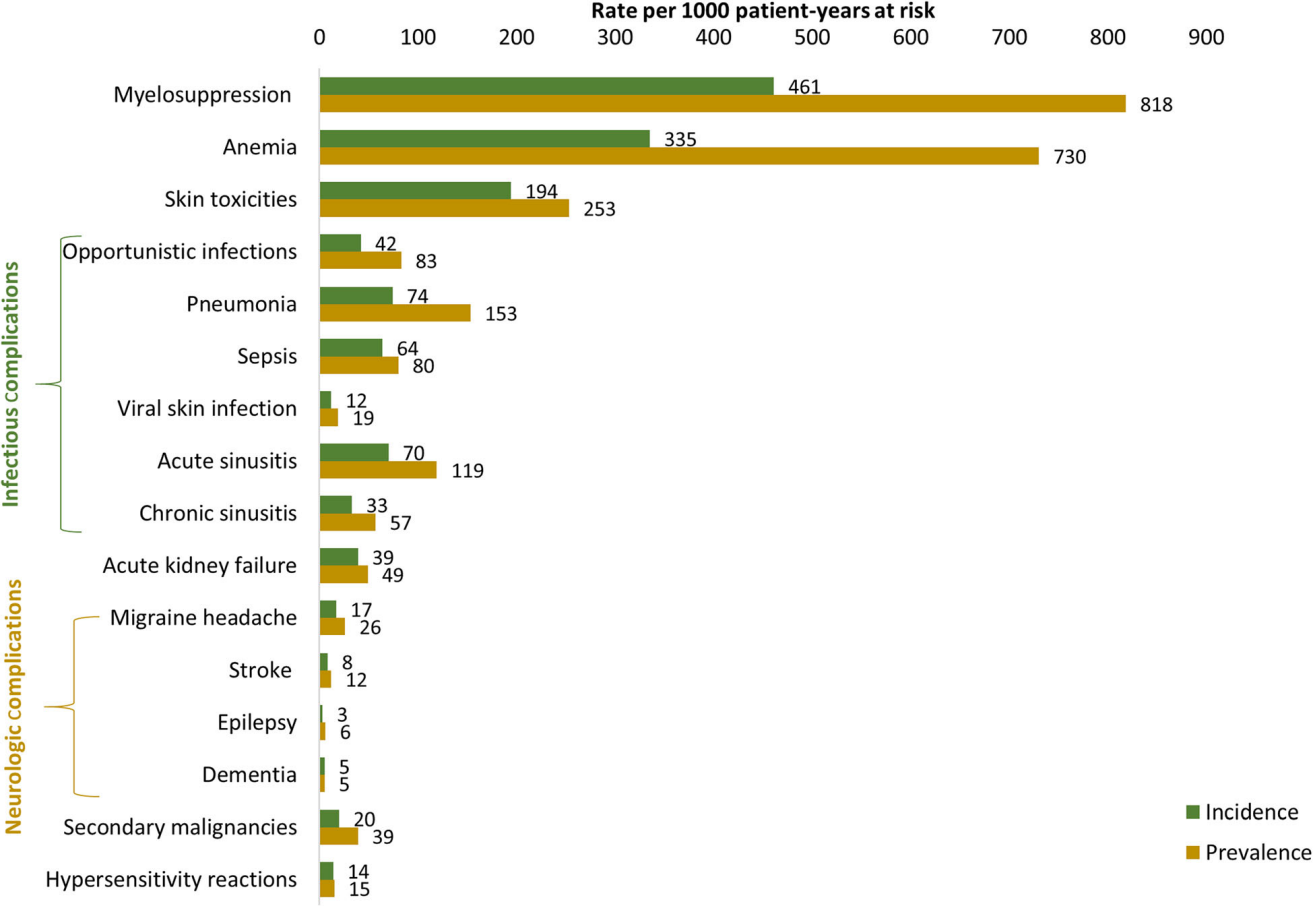
Incidence and prevalence of AEs over the 12-month follow-up period among PNA-treated patients with HCL. AEs were identified at any position (primary, secondary) on the claim. *AE* adverse event, *HCL* hairy cell leukemia, *PNA* purine nucleoside analog

Over the 12-month follow-up period, 87.2% of the PNA-treated patients with HCL developed ≥1 AE. The PNA-related AEs with the highest incidence and prevalence were myelosuppression (incidence, 461; prevalence, 818 per 1000 patient-years), anemia (incidence, 335; prevalence, 730 per 1000 patient-years), and skin toxicities (incidence, 194; prevalence, 253 per 1000 patient-years) (Fig. 2). Infectious complications including OIs, pneumonia, sepsis, and acute sinusitis were observed at a higher rate than neurologic complications, as depicted in Fig. 2. The incidence and prevalence of overall infectious complications was 235 and 393 per 1000 patient-years, respectively.

### Outcomes among patients with and without myelosuppression

**Fig. 3:**
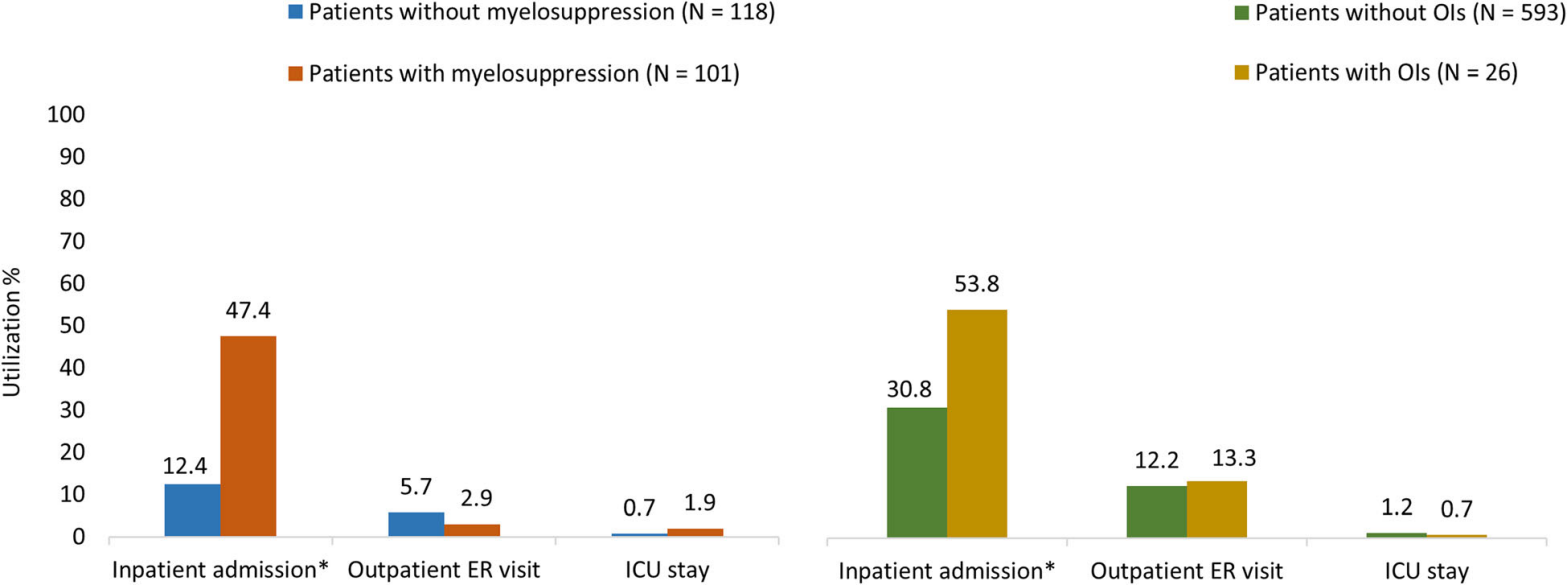
All-cause HCRU during the 12-month follow-up period. *ER* emergency department/room, *ICU* intensive care unit, *OI* opportunistic infection. *Significant at *P* < .05

**Fig. 4:**
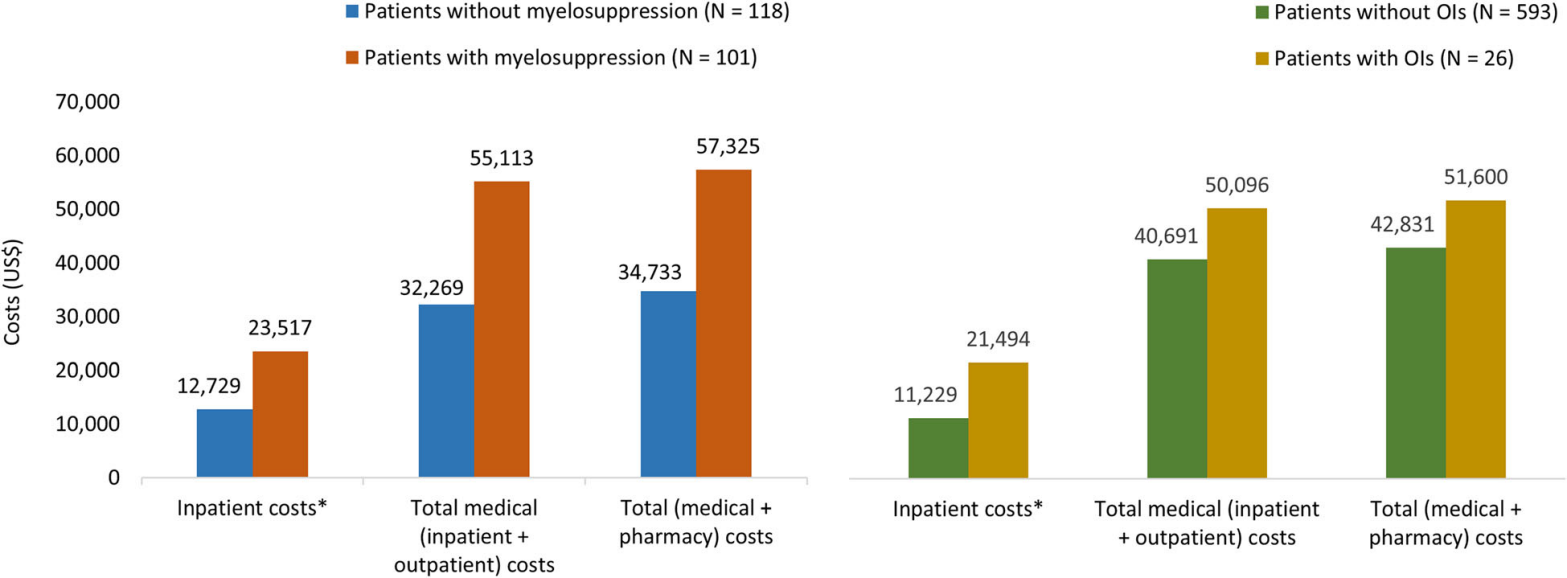
All-cause costs during the 12-month follow-up period. *Significant at *P* < .05

GLM-adjusted results for all-cause HCRU indicated that a greater proportion of patients who developed myelosuppression were hospitalized (47.4% vs 12.4%; *P* < .0001) as compared with those who did not (Fig. 3). Patients who developed myelosuppression had longer all-cause inpatient length of stay (LOS) (3.4 vs 0.8 days; *P* = .001) and a higher number of mean all-cause inpatients visits (3.1 vs 0.8; *P* = .001) as compared with those who did not (Additional file 3: Table S3). Patients who developed myelosuppression incurred significantly higher mean all-cause inpatient costs ($23,517 vs $12,729; *P* = .011) as compared with their counterparts (Fig. 4); however, the mean all-cause outpatient office costs were comparable for both groups ($14,231 vs $11,334; *P* = .144) (Additional file 3: Table S3). The estimated all-cause total medical (inpatient and outpatient) costs ($55,113 vs $32,269; *P* < .0001) and total (medical and pharmacy) costs ($57,325 vs $34,733; *P* = .001) were significantly higher for the patients who developed myelosuppression as compared with those who did not (Fig. 4). Inpatient cost was the major driver of the total health care costs.

### Outcomes among patients with and without OIs

GLM-adjusted results for all-cause HCRU indicated that a greater proportion of patients who developed OIs were hospitalized (53.8% vs 30.8%; *P* = .025) as compared with those who did not develop OIs (Fig. 3). Patients who developed OIs incurred significantly higher mean all-cause inpatient costs ($21,494 vs $11,229; *P* < .0001) and outpatient office costs ($20,398 vs $12,841; *P* = .028) but lower emergency department/room (ER) costs ($156 vs $231; *P* = .042) as compared with those who did not develop OIs (Fig. 4 and Additional file 4: Table S4). The estimated all-cause total medical (inpatient and outpatient) costs ($50,096 vs $40,691; *P* = .321) and total (medical and pharmacy) costs ($51,600 vs $42,831; *P* = .374) were higher for the patients who developed OIs compared with their counterparts; however, the difference was not statistically significant (Fig. 4).

## Discussion

Patients treated with PNA as monotherapy or in combination with rituximab were included in this analysis; 152 patients treated with rituximab only were not included. The use of PNAs is associated with increased toxicity and AEs based on the data from clinical trials [3, 16, 20–22]; however, there is a paucity of data on the real-world incidence, prevalence, and economic burden of AEs. Hence, this retrospective claims-based study examined the incidence and prevalence of AEs associated with PNA use, as well as HCRU and costs among the sub-cohorts of patients experiencing myelosuppression or OIs.

The findings on incidence and prevalence in our dataset revealed that the most common AEs among PNA-treated patients with HCL were myelosuppression (461 per 1000 patient-years) followed by anemia (335 per 1000 patient-years) and infectious complications (235 per 1000 patient-years). Our findings are in agreement with the information disclosed on the US Food and Drug Administration (FDA) label for PNA therapies. According to the FDA label for cladribine, during the first month of the HCL clinical trials, 54 of 196 patients (28%) exhibited documented evidence of infection. Myelosuppression was frequently observed during the first month after starting treatment, and severe anemia (hemoglobin < 8.5 g/dL) developed in 37% of patients [20]. A report by Sigal et al. on the activity of cladribine also identified the same common cladribine toxicities [21]. The FDA label for pentostatin states that in the clinical trials, 63% of patients with HCL treated with pentostatin had nausea/vomiting and 46% had a fever. Additionally, anemia and infection occurred among 8% and 7% of the patients, respectively [22].

Furthermore, our study revealed that a substantial number of PNA-treated patients with HCL developed OIs, with an incidence and prevalence of 42 and 83 per 1000 patient-years, respectively. There have been reports in the literature suggesting that patients with HCL are likely to suffer from infectious complications and treatment-related mortality [8, 12]. However, owing to the nature of the dataset in this study, information regarding mortality is not currently available. There is a need for future research focusing on PNA treatment-related mortality, given that patients who respond to PNA treatment are likely to have similar survival rates when compared to age-matched counterparts [23].

The current study also examined the HCRU and costs among the sub-cohorts of patients with and without myelosuppression and OIs. To date, there has been no prior study that has examined such outcomes specifically among patients experiencing myelosuppression or OIs while receiving PNA therapies. We discovered that a greater proportion of PNA-treated patients with HCL who developed myelosuppression were hospitalized (47.4% vs 12.4%; *P* < .0001) and had longer mean inpatient LOS (3.4 vs 0.8 days; *P* = .001) as compared with those who did not develop myelosuppression. Consequently, these patients incurred higher (on average $22,592 more) total costs than their counterparts. Similarly, a greater proportion of PNA-treated patients with HCL who developed OIs were hospitalized (53.8% vs 30.8%; *P* = .025) and had higher hospital stay costs ($21,494 vs $11,229; *P* < .0001) as compared with those who did not develop OIs. The total health care costs were higher for those who developed OIs as compared with those who did not; however, the data lacked statistical significance ($51,600 vs $42,831; *P* = .374). This may be attributed to the smaller size of this sub-cohort of patients who developed OIs (*n* = 26), which indicates a need to further explore this finding in a larger sample.

Apart from the findings during the follow-up period, a few notable observations were also made during the baseline period. Our study showed that during the baseline period, patients who developed OIs were significantly older than those who did not develop OIs. Further examination of the relationship between age and outcomes of interest revealed that age was significantly associated with outpatient ER visits, intensive care unit stays, and the number of inpatient, outpatient, ER, other outpatient, intensive care unit, and pharmacy visits. Furthermore, our study also found that a significantly greater proportion of patients who developed OIs had diabetes in the baseline period as compared with those who did not develop OIs (26.9% vs 10.3%; *P* = .008), possibly indicating that diabetes portends a high risk for OI occurrence and that clinicians should be vigilant regarding this potential complication.

Our study findings should be viewed in the context of claims data limitations. While claims data are extremely valuable for the efficient and effective examination of health care outcomes, they pose unique research challenges. Claims data are mainly collected for administrative rather than research purposes. Therefore, claims-based analyses may be subject to inherent limitations of the source administrative claims data, such as coding errors or diagnoses entered for administrative processing rather than clinical completeness. Moreover, the presence of a diagnosis code on a medical claim is not a positive indication of the presence of the disease, as the diagnosis code may have been incorrectly coded or included as rule-out criteria rather than the actual disease. Furthermore, certain information is not readily available in claims data that could influence study outcomes, such as clinical and laboratory parameters, rendering analysis susceptible to potential residual bias. In addition, it is important to recognize that utilization results gained from claims analysis apply only to the insured population. Lastly, and specific to this study, with claims data it is difficult to determine whether the identified HCL diagnosis is the HCL variant or the classical HCL, because both use the same ICD-9/10-CM codes. Also, dose information had not been assessed in this study because PNAs have weight-based dosing that is not available in claims data.

## Conclusions

Notwithstanding these limitations, to the best of our knowledge, this is the first claims-based study to examine the burden of AEs among PNA-treated patients with HCL. The study identified myelosuppression and OIs as principal drivers of economic burden. A substantial proportion of patients developed AEs, with myelosuppression being the highest-incident AE, followed by anemia and skin toxicities. The incidence and prevalence of infectious complications were also notable, with pneumonia being the most common. PNA-treated patients with HCL who developed myelosuppression or OIs had higher HCRU and costs as compared with those who did not develop either condition. These findings indicate a need for larger studies evaluating the outcomes of HCL-diagnosed patients treated with approved therapies as well as associated short- and long-term toxicities. Future studies should focus on better risk stratification of patients who were vulnerable to develop myelosuppression and OIs. Future investigation in this area should be employed to identify strategies to prolong long-term survival and enable health care professionals and other stakeholders to better manage costs among patients diagnosed with HCL.

## Supplementary information

**Additional file 1: Table S1.** Baseline individual comorbidities and corresponding ICD-9-CM codes.

**Additional file 2: Table S2.** List of PNA-related adverse events and corresponding ICD-9-CM Codes.

**Additional file 3: Table S3.** GLM-adjusted follow-up outcomes among myelosuppression sub-cohorts.

**Additional file 4: Table S4.** GLM-adjusted follow-up outcomes among opportunistic infection sub-cohorts.

## Data Availability

The raw insurance claims data used for this study originate from Medicare data, which are available from the Centers for Medicare and Medicaid through ResDAC (https://www.resdac.org/).

## Abbreviations

AE: Adverse event
CR: Complete remission
FDA: Food and Drug Administration
GLM: Generalized linear model
HCL: Hairy cell leukemia
HCRU: Health care resource utilization
ICD-9/10-CM: International Classification of Diseases, Ninth/Tenth Revision, Clinical Modification
LOS: Length of stay
OI: Opportunistic infection
PNA: Purine nucleoside analog
Quan-CCI: Quan-Charlson comorbidity index
