# Supplementary material for: Adverse event rates and economic burden associated with purine nucleoside analogs in patients with hairy cell leukemia: a US population-retrospective claims analysis
Source: Orphanet J Rare Dis. 2020 Feb 13;15:47. doi: 10.1186/s13023-020-1325-9 (PMC7020358; doi:10.1186/s13023-020-1325-9)
Supplement: Supplementary file 1 — Additional file 1: Table S1. Baseline individual comorbidities and corresponding ICD-9-CM codes. [file 13023_2020_1325_MOESM1_ESM.docx]

**Supplementary information**

| **Supplementary Table S1: Baseline individual comorbidities and corresponding ICD-9-CM codes** | |
| --- | --- |
| **Baseline comorbidities** | **ICD-9-CM codes** |
| Infectious complications |  |
| Pneumonia | 480–483, 485–486, 487.0 |
| Viral skin infections | 686.8 (Other specified local infections of skin and subcutaneous tissue) |
|  | 686.9 (Unspecified local infection of skin and subcutaneous tissue) |
| Sepsis | 038.x, 995.91, 995.92,659.3x |
| Opportunistic infections | 011.x (Pulmonary tuberculosis) |
|  | 031.x (Atypical mycobacteria) |
|  | 117.5 (Cryptococcosis) |
|  | 117.3 (Aspergillosis) |
|  | 115.x (Histoplasmosis) |
|  | 027.0 (Listeriosis) |
|  | 085.x (Leishmaniasis) |
|  | 136.3 (Pneumocystis jiroveci pneumonia) |
|  | 370.x (Keratitis) |
|  | 110.1 (Onychomycosis) |
|  | 567.0, 567.2x, 567.8x, 567.9 (Peritonitis) |
|  | 117.9 (other and unspecified mycoses) (Fungemia) |
|  | 360.0 (Endophthalmitis) |
|  | 711.0x (Septic Arthritis, Pyogenic Arthritis) |
|  | 730.xx (Osteomyelitis) |
| Acute sinusitis | 461 |
| Chronic sinusitis | 473 |
| Abdominal pain | 789 |
| Adenopathy | 683, 785.6, 289.2, 289.3 |
| Diabetes | 250-250.9 |
| Fatigue | 780.7x |
| Hypertension | 401-405.99, 997.91 |
| Liver disease | 570, 571 (Liver disease) |
| Liver enlargement | 789.1 (Hepatomegaly) |
| Myocardial infraction | 410, 412 |
| Splenomegaly | 789.2 |
| Kidney (renal) failure | 584.xx (acute), 585 .xx (chronic), 586.xx (unspecified) |
|  |  |
| *All ICD-9-CM codes were mapped to ICD-10-CM codes based on the General Equivalence Mappings (GEMs) published by Centers for Medicare and Medicaid* | |
| *Services (CMS). Available at https://www.cms.gov/Medicare/Coding/ICD10/index.html* | |
