# Supplementary material for: Adverse event rates and economic burden associated with purine nucleoside analogs in patients with hairy cell leukemia: a US population-retrospective claims analysis
Source: Orphanet J Rare Dis. 2020 Feb 13;15:47. doi: 10.1186/s13023-020-1325-9 (PMC7020358; doi:10.1186/s13023-020-1325-9)
Supplement: Supplementary file 2 — Additional file 2: Table S2. List of PNA-related adverse events and corresponding ICD-9-CM Codes. [file 13023_2020_1325_MOESM2_ESM.docx]

| **Supplementary Table S2: List of PNA-related adverse events and corresponding ICD-9-CM Codes** | | | |
| --- | --- | --- | --- |
| **Adverse events** | **ICD-9-CM codes** | |  |
| **Skin toxicities** | 373.0x, 528.5, 681.x, 682.x, 693.0, 693.8, 693.9, 782.1 | |  |
| **Hypersensitivity reactions** | 995.29 | |  |
| **Secondary malignancies** | 197-199 | |  |
| **Infectious complications** |  | |  |
| Pneumonia | 480–483, 485–486, 487.0 | |  |
| Viral skin infection | 686.8 (Other specified local infections of skin and subcutaneous tissue) | |  |
|  | 686.9 (Unspecified local infection of skin and subcutaneous tissue) | |  |
| Sepsis | 038.x, 995.91, 995.92,659.3x | |  |
| Opportunistic infections | 011.x (Pulmonary tuberculosis) | |  |
|  | 031.x (Atypical mycobacteria) | |  |
|  | 117.5 (Cryptococcosis) | |  |
|  | 117.3 (Aspergillosis) | |  |
|  | 115.x (Histoplasmosis) | |  |
|  | 027.0 (Listeriosis) | |  |
|  | 085.x (Leishmaniasis) | |  |
|  | 136.3 (Pneumocystis jiroveci pneumonia) | |  |
|  | 370.x (Keratitis) | |  |
|  | 110.1 (Onychomycosis) | |  |
|  | 567.0, 567.2x, 567.8x, 567.9 (Peritonitis) | |  |
|  | 117.9 (other and unspecified mycoses) (Fungemia) | |  |
|  | 360.0 (Endophthalmitis) | |  |
|  | 711.0x (Septic Arthritis, Pyogenic Arthritis) | |  |
|  | 730.xx (Osteomyelitis) | |  |
| Acute sinusitis | 461 | |  |
| Chronic sinusitis | 473 | |  |
| **Myelosuppression** | 288 (neutropenia), 287.3–287.5 (Thrombocytopenia), 289.9 | |  |
| **Anemia** | 280-285.99 | |  |
| **Neurological complications** |  | |  |
| Amyotrophic lateral sclerosis (ALS) | 335.2 | |  |
| Stroke | 430–431 (Subarachnoid or intracerebral hemorrhage) | |  |
|  | 433.11, 433.21, 433.81, 433.91, 434.01, 434.11, 434.91 (Cerebral infarction) | |  |
|  | 436 (Acute cerebrovascular disease) | |  |
| Dementia | 290.00, 290.20, 290.40-290.42, 291.2, 294.10, 294.11, 294.20 | |  |
| Epilepsy | 345.xx | |  |
| Migraine headache | | 346 |  |
| Parkinson disease | | 332.xx |  |
| Neurological toxicity | | 344.1x (Paraparesis) |  |
|  |  | 344.0x (Quadraparesis) |  |
| **Acute kidney failure** | | 584.xx (acute) |  |
|  | |  |  |
| *All ICD-9-CM codes were mapped to ICD-10 codes based on the General Equivalence Mappings (GEMs) published by Centers for Medicare and Medicaid Services (CMS). Available at* [*https://www.cms.gov/Medicare/Coding/ICD10/index.html*](https://www.cms.gov/Medicare/Coding/ICD10/index.html) | | |  |
