# Supplementary material for: Adverse event rates and economic burden associated with purine nucleoside analogs in patients with hairy cell leukemia: a US population-retrospective claims analysis
Source: Orphanet J Rare Dis. 2020 Feb 13;15:47. doi: 10.1186/s13023-020-1325-9 (PMC7020358; doi:10.1186/s13023-020-1325-9)
Supplement: Supplementary file 3 — Additional file 3: Table S3. GLM-adjusted follow-up outcomes among myelosuppression sub-cohorts. [file 13023_2020_1325_MOESM3_ESM.docx]

**Supplementary Table S3: GLM-adjusted follow-up outcomes among myelosuppression sub-cohorts**

| **12-month follow-up outcomes** | **Sub-cohort with no myelosuppression in baseline (N=219)** | | |
| --- | --- | --- | --- |
|  | **Patients without myelosuppression (N=118)** | **Patients with myelosuppression (N=101)** | **P-value** |
|  | **%/Mean** | **%/Mean** |  |
| **Concomitant medications** |  |  |  |
| Acyclovir | 3.0% | 3.2% | 0.936 |
| Valacyclovir | 0.3% | 1.0% | 0.065 |
| Famciclovir | 0.0% | 0.0% | 0.854 |
| Atovaquone | 0.0% | 0.0% | 1.000 |
| Pentamidine | 0.0% | 0.0% | N/A |
| Antibiotics (oral and IV) | 100.0% | 100.0% | N/A |
| **All-cause health care resource utilization** |  |  |  |
| **Number of patients with any visit** |  |  |  |
| Inpatient admission | 12.4% | 47.4% | **<.0001*** |
| Outpatient ER visit | 5.7% | 2.9% | 0.109 |
| Outpatient office visit | 100.0% | 100.0% | 0.621 |
| Other outpatient visit | 100.0% | 100.0% | 0.961 |
| ICU stay | 0.7% | 1.9% | 0.068 |
| Pharmacy visit | 99.6% | 99.4% | 0.575 |
| **Number of visits [mean]** |  |  |  |
| Inpatient length of stay (in days) | 0.8 | 3.4 | **0.001*** |
| Inpatient visits | 0.8 | 3.1 | **0.001*** |
| Outpatient ER visits | 0.1 | 0.1 | 0.148 |
| Outpatient office visits | 19.9 | 22.7 | 0.134 |
| Other outpatient visits | 12.1 | 13.5 | 0.365 |
| ICU stays | 0.0 | 0.0 | 0.332 |
| Pharmacy visits | 10.2 | 9.3 | 0.614 |
| **All-cause health care costs [mean]** |  |  |  |
| Inpatient costs | $12 729 | $23 517 | **0.011*** |
| Outpatient ER costs | $282 | $356 | 0.627 |
| Outpatient office costs | $11 334 | $14 231 | 0.144 |
| Other outpatient costs | $12 245 | $15 760 | 0.110 |
| ICU^costs** | N/A | N/A | N/A |
| Pharmacy costs | $3 748 | $2 299 | 0.132 |
| Total medical (inpatient + outpatient) costs | $32 269 | $55 113 | **<.0001*** |
| Total (medical + pharmacy) costs | $34 733 | $57 325 | **0.001*** |
| ** significant at p<0.05* |  |  | |
| *^ ICU cost is a sub-set of inpatient costs* |  |  | |
| ***Model did not converge for ICU cost* |  |  | |
