# Supplementary material for: Adverse event rates and economic burden associated with purine nucleoside analogs in patients with hairy cell leukemia: a US population-retrospective claims analysis
Source: Orphanet J Rare Dis. 2020 Feb 13;15:47. doi: 10.1186/s13023-020-1325-9 (PMC7020358; doi:10.1186/s13023-020-1325-9)
Supplement: Supplementary file 4 — Additional file 4: Table S4. GLM-adjusted follow-up outcomes among opportunistic infection sub-cohorts. [file 13023_2020_1325_MOESM4_ESM.docx]

**Supplementary Table S4: GLM-adjusted follow-up outcomes among opportunistic infection sub-cohorts**

| **12-month follow-up outcomes** | **Sub-cohort with no opportunistic infection in the baseline (N=619)** | | |
| --- | --- | --- | --- |
|  | **Patients without opportunistic infection (N=593)** | **Patients with opportunistic infection (N=26)** | **p-value** |
|  | **%/Mean** | **%/Mean** |  |
| **Concomitant medications** |  |  |  |
| Acyclovir | 5.7% | 8.0% | 0.524 |
| Valacyclovir | 4.5% | 9.1% | 0.231 |
| Famciclovir | 0.0% | 0.0% | 1.000 |
| Atovaquone | 0.0% | 0.0% | 0.086 |
| Pentamidine | 0.0% | 0.0% | N/A |
| Antibiotics (oral and IV) | 100.0% | 100.0% | N/A |
| **All-cause health care resource utilization** |  |  |  |
| **Number of patients with any visit** |  |  |  |
| Inpatient admission | 30.8% | 53.8% | **0.025*** |
| Outpatient ER visit | 12.2% | 13.3% | 0.832 |
| Outpatient office visit | 100.0% | 100.0% | 1.000 |
| Other outpatient visit | 100.0% | 100.0% | 0.959 |
| ICU stay | 1.2% | 0.7% | 0.514 |
| Pharmacy visit | 99.6% | 99.5% | 0.825 |
| **Number of visits [mean]** |  |  |  |
| Inpatient length of stay (in days) | 2.4 | 5.5 | 0.132 |
| Inpatient visits | 2.2 | 5.0 | 0.114 |
| Outpatient ER visits | 0.1 | 0.1 | 0.562 |
| Outpatient office visits | 21.1 | 26.2 | 0.086 |
| Other outpatient visits | 13.5 | 13.3 | 0.920 |
| ICU stays | 0.0 | 0.0 | 0.394 |
| Pharmacy visits | 11.1 | 10.1 | 0.687 |
| **All-cause health care costs [mean]** |  |  |  |
| Inpatient costs | $11 229 | $21 494 | **<.0001*** |
| Outpatient ER costs | $231 | $156 | **0.042*** |
| Outpatient office costs | $12 841 | $20 398 | **0.028*** |
| Other outpatient costs | $16 367 | $12 867 | 0.249 |
| ICU^ costs | $5 239 | $2 806 | 0.820 |
| Pharmacy costs | $2 305 | $2 589 | 0.565 |
| Total medical (inpatient + outpatient) costs | $40 691 | $50 096 | 0.321 |
| Total (medical + pharmacy) costs | $42 831 | $51 600 | 0.374 |

* significant at p<0.05

^ ICU cost is a sub-set of inpatient costs.

Other outpatient includes services carried out in hospices, urgent care facility, independent clinics, rural health clinics, independent laboratory, etc.

Variables included in the GLM were comprised of age, sex, US region, Quan-Charlson comorbidity index (CCI) score, baseline individual comorbidities, baseline heme-related diagnoses, baseline medications, and baseline all-cause total health care costs.
